# Supplementary material for: Ionizing radiation induces cells with past caspase activity that contribute to the adult organ in Drosophila and show reduced Loss of Heterozygosity
Source: Cell Death Discov. 2024 Jan 5;10:6. doi: 10.1038/s41420-023-01769-4 (PMC10770159; doi:10.1038/s41420-023-01769-4)
Supplement: Supplementary file 1 — Supplemental Data [file 41420_2023_1769_MOESM1_ESM.pdf]

Supplemental Figure 1

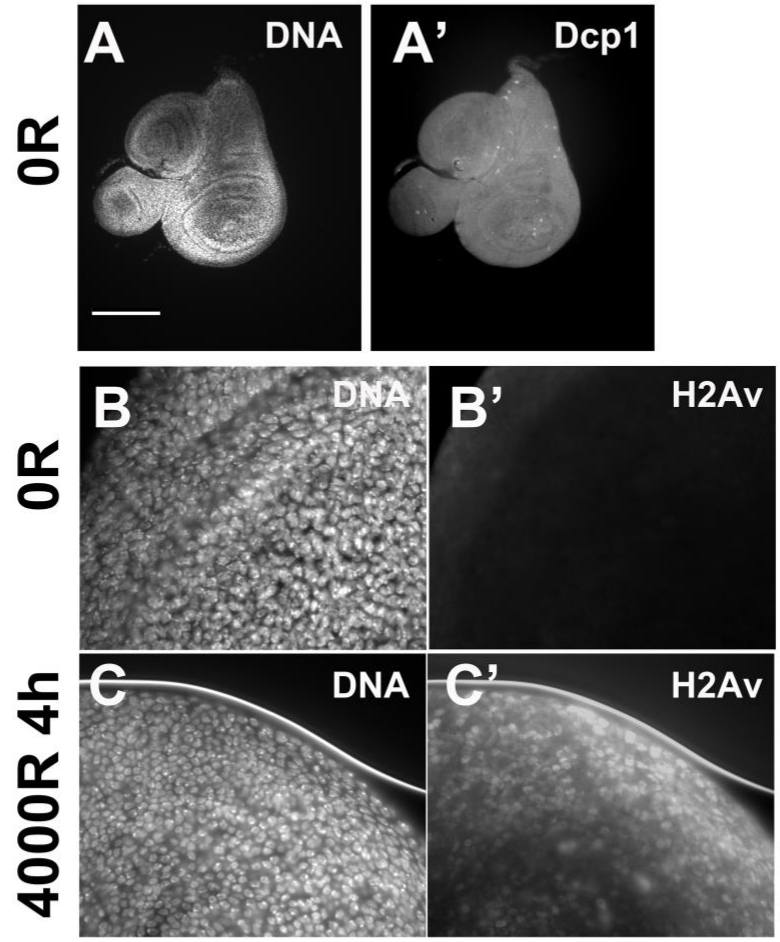

Figure S1. Caspase cleavage without IR and DNA double strand breaks with and without IR.
Larvae of the genotype CasExpress/G-trace; GAL80^{ts}/+ were treated as in Fig. 1A where T=26°C. Wing discs were dissected at 4h after exposure to 0 or 4000R of X-rays, fixed, and stained for DNA and with antibodies to cleaved Dcp1 (A-A') or γ-H2Av (B-C'). The scale bar = 120 microns in A and 24 microns in B-C.

Supplemental Table 1. Fly stocks used

Source (BL = Bloomington Stock Center)	Used in Fig.	Genotype
BL28280 ‘G-trace’	all	P{w[+mC]=UAS-RedStinger}4, P{w[+mC]=UAS-FLP.D}JD1, P{w[+mC]=Ubi-p63E(FRT.STOP)Stinger}9F6
BL65419	all	P{w[+mC]=Ubi-CasExpress}attP40
BL7017	all	tub-GAL80^{ts} on chromosome III
BL65420	1	P{w[+mC]=Ubi-CasExpress.DQVA}attP40
Ref 12	1	Caspase Tracker
BL1576	3	Df(3L)H99/TM6B Tb
BL5823	3	w1118 P{UAS-rpr.C}27 (UAS-rpr on the X chromosome)
Ref 31	3	UAS-hid, UAS-rpr on the X chromosome
BL5073	3	w[*]; P{w[+mC]=UAS-p35.H}BH2
Ref 33	3	UAS-Dronc^{DN} on chromosome III
BL33623	3	UAS- w RNAi on chromosome III
Ref 32	5	UAS-Wg on chromosome III
BL7225	5	w[*]; Kr[If-1]/CyO; P{w[+mC]=UAS-Axn.GFP}3 (UAS-Axin on chromosome III)
BL26674	5	y[1] w[*]; P{w[+mC]=UAS-Dl::N.DeltaECN}B2a3 (constitutively active N on Ch. III)
BL26672	5	w[*]; P{w[+mC]=UAS-mamN}3 (dominant negative Mastermind on Ch. III)
BL64195	5	w[*]; P{w[+mC]=UAS-Ras85D.V12}TL1 (constitutively active Ras on Ch. III)
BL549	6	mwh[1]
